# A review of uphill and downhill running: biomechanics, physiology and modulating factors

**DOI:** 10.3389/fbioe.2025.1690023

**Published:** 2025-10-24

**Authors:** Zeyu Lu, Bokai Suo, Liqin Deng, Jichao Wang, Weijie Fu, Yunjian Zhong, Jing Jin

**Affiliations:** ^1^ Key Laboratory of Exercise and Health Sciences of Ministry of Education, Shanghai University of Sport, Shanghai, China; ^2^ School of Intelligent Sports Engineering, Shanghai University of Sport, Shanghai, China; ^3^ College of Physical Education, Nanchang University, Nanchang, China; ^4^ School of Psychology, Shanghai University of Sport, Shanghai, China

**Keywords:** graded running, uphill running, downhill running, biomechanics, physiology

## Abstract

Graded running imposes distinct biomechanical and physiological demands compared with level running, which influences performance outcomes and injury risk. Uphill running requires great propulsion and energy expenditure, which results in increased oxygen consumption and cardiovascular burden. By contrast, downhill running relies heavily on eccentric muscle contractions, which show an association with great fatigue and a high risk of injury. This review aims to summarize biomechanical and physiological adaptations to uphill and downhill running and evaluate the mechanisms underlying the interaction of modulating factors (footwear, foot strike patterns, individual characteristics, pacing strategies, cadence and running speed) with slope. Based on current evidence, these factors affect mechanical loading, energy cost and neuromuscular responses during uphill and downhill running. Footwear design, especially carbon-fiber plate shoes, can reduce metabolic cost during uphill and downhill running. Forefoot striking reduces peak impact forces compared to rearfoot striking, especially in downhill running, helping to lower impact on the lower limbs. Individual factors, like training level, influence the effectiveness of these factors, with experienced runners adapting better to slopes. Additionally, adjusting cadence can reduce per-step load and energy expenditure during uphill and downhill running. Although considerable progress has been made, further research still necessitates the exploration of graded running in real-world environments, improvement of training methods, clarification of the interactions between various factors and slope and optimization of methods to prevent injuries.

## 1 Introduction

Running is a basic human locomotion mode, and studies have widely focused on its biomechanical adaptations, physiological demands, performance determinants and associated injury risks (Willwacher et al., 2022; Llanos-Lagos et al., 2024). Although most of basic research has focused on running on flat terrain, the growing popularity of activities, such as trail running (Scheer et al., 2020), has highlighted the need for an in-depth understanding of running on varied surfaces. Importantly, graded terrain is common in trails: urban road races also include sustained uphill/downhill sections (e.g., Boston Marathon and Hakone Ekiden courses), which many runners perceive as challenging despite their smooth surfaces. Running uphill and downhill presents biomechanical, neuromuscular and physiological demands that considerably differ from those of level running (LR) and affect gait patterns, muscle activation strategies, energy expenditure and tissue loading (Khassetarash et al., 2020; Vernillo et al., 2017; Vernillo et al., 2020b). Researchers and practitioners working with runners across trail and road settings should gain a detailed understanding of the biomechanics and physiological responses of uphill running (UR) and downhill running (DR).

Meanwhile, research progress has witnessed the proposal of several candidate factors for the modulation of the biomechanics and physiology of graded running. Rather than assuming uniform effects, this review evaluated whether and to what extent the factors (e.g., footwear, foot strike pattern, individual runner characteristics, pacing strategy, cadence and running speed) alter slope-induced responses and injury-relevant loads (Besson et al., 2023; Johnson et al., 2022; Khassetarash et al., 2023; Lussiana et al., 2013; Sabater Pastor et al., 2023; Selvakumar et al., 2023; Vernillo et al., 2024; Vincent et al., 2019). These variables may also interact with the surface slope to regulate biomechanical and physiological responses during graded running. Yet, current evidence on their combined effects remains inconsistent. Accordingly, this review summarised existing studies to determine how slope-induced changes in spatiotemporal parameters, ground reaction force (GRF), joint mechanics, muscle activity and metabolic cost are further influenced by footwear, foot strike pattern, individual characteristics, pacing strategy, cadence and running speed. Clarification of these multifactor interactions will lead to improved inform training prescriptions, enhanced performance and minimised risk of injury.

This review aimed to summarise current evidence on the biomechanical and physiological adaptations during UR and DR and clarify the mechanism underlying the influence of modulating factors, such as footwear, foot strike pattern, individual characteristics, pacing strategy, cadence and running speed, on performance and injury risk. Article search was conducted using keywords, such as *level*, *uphill*, *downhill*, *incline*, *decline*, *grade*, *gradient*, *slope*, *running*, *biomechanics* and *physiology* in the databases of PubMed, Web of Science and Google Scholar. The time frame for the search was conducted up to July 2025. Studies were included if they focused on the biomechanics and physiology of graded running, and excluded if they did not involve the topic of this review or were not published in peer-reviewed journals. To optimise the search process, in addition to electronic database searches, we also reviewed references in the reference catalogue of relevant articles. Included articles must be complete and accessible. Unpublished or inaccessible works were generally excluded from selection. Furthermore, only English-language literature was considered to ensure broader accessibility and consistency within the academic community. Since previous reviews have summarized some relevant articles, this review aims to present new developments and the latest biomechanical and physiological adaptation mechanisms in this field. Therefore, some duplicative articles have not been cited. In literature, the terms “slope” and “gradient” have been used interchangeably, and in this document, unless explicitly stated otherwise, both terms refer to movements on surfaces of different inclinations.

## 2 Biomechanical adaptations to graded running

This section summarises the intrinsic kinematic, kinetic and energetic changes produced solely by surface gradient (uphill and downhill). Section 4 analyses separately the corresponding modulating factors.

**FIGURE 1:**
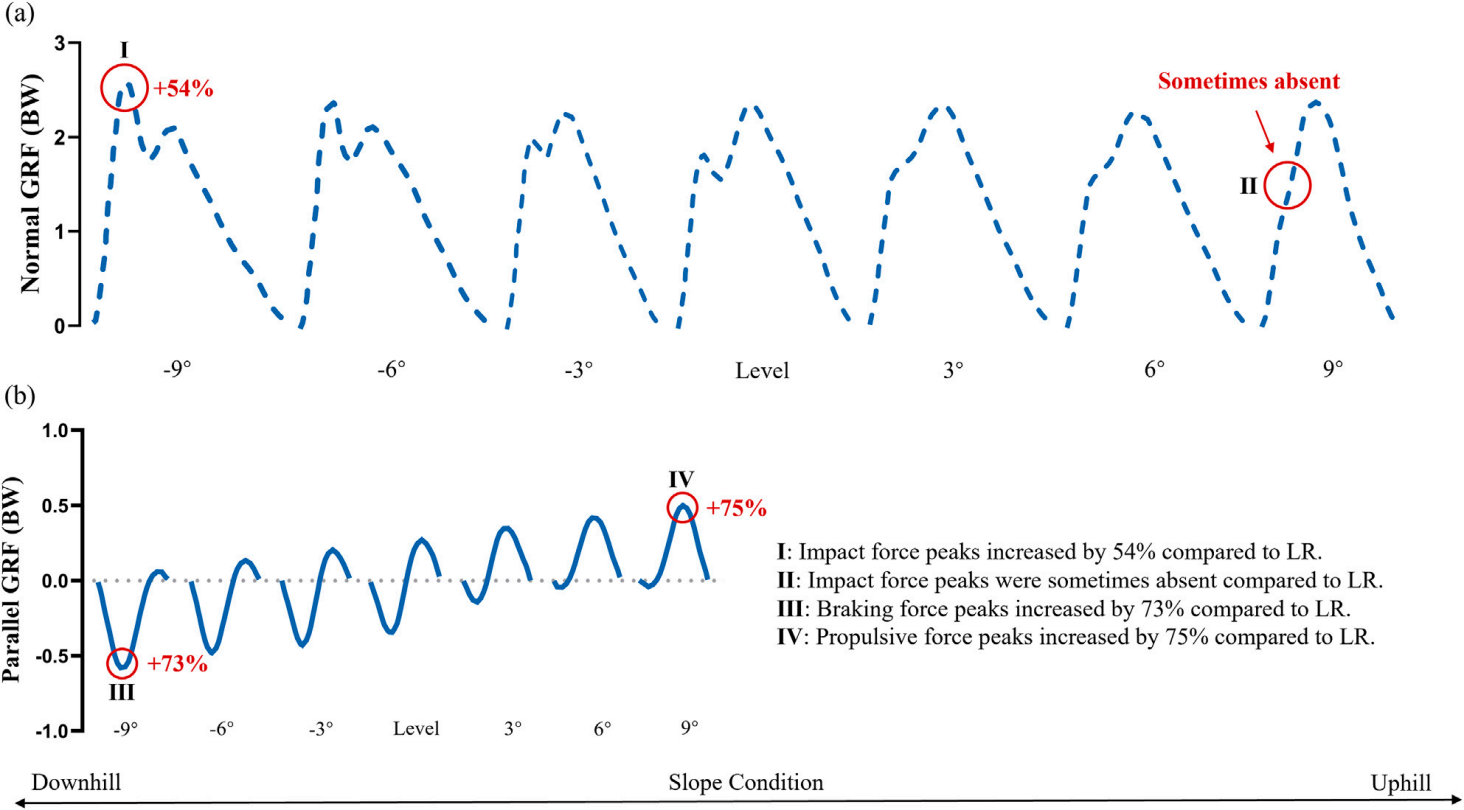
Ground reaction forces (GRFs) normalized as percentage of body weight (BW) in different gradient conditions during treadmill running at 3 m/s: **(a)** normal GRF; **(b)** parallel GRF. Adapted and based on data from Gottschall and Kram (2005); LR: Level Running.

Running on inclined or declined surfaces necessitates fundamental adjustments to gait mechanics compared with level ground. These adaptations are primarily under the influence of the altered gravitational forces acting on the runner and require different strategies for force generation (propulsive force) and absorption (braking force) (Bontemps et al., 2025; Bontemps et al., 2020; Coratella et al., 2024; Giandolini et al., 2017; Giandolini et al., 2016b; Khassetarash et al., 2020; Vernillo et al., 2017; Vernillo et al., 2020b). The reviewed literature explored changes across various biomechanical parameters, including spatial–temporal parameters, GRF (Figure 1), joint kinematics and kinetics (Figure 3).

### 2.1 Spatial–temporal parameters

Spatial–temporal parameters, such as cadence (step frequency), stride length, contact time, aerial time and duty factor (the ratio of stance time to stride time), were tremendously influenced by surface gradient and speed (Genitrini et al., 2024; Padulo et al., 2012; van Oeveren et al., 2024; Vernillo et al., 2020b). During UR, runners tended to increase their cadence and decrease their stride length compared with LR at the same speed (Padulo et al., 2012; Vernillo et al., 2017; Vernillo et al., 2020b). During treadmill UR at 7% slope, the cadence increased by 4.5%, and the stride length and aerial time decreased by 4.3% and 13.7% respectively at the speed of 4.17 m/s (Padulo et al., 2013). This strategy, particularly the reliance on increasing cadence rather than the stride length to increase speed during UR, differed from the typical LR approach, where both parameters contributed to speed increases (Khassetarash et al., 2020; Padulo et al., 2012). UR also resulted in a shorter swing or aerial phase and a greater duty factor, indicating that a larger proportion of the stride cycle was allotted to contact time (Khassetarash et al., 2020; Vernillo et al., 2017; Vernillo et al., 2020b). The increased proportion of contact time might allow runners to apply greater force against the inclined surface, facilitating vertical propulsion against gravity.

Conversely, DR was characterized by a tendency toward increased aerial time and reduced cadence, and decreased duty factor compared with LR (Vernillo et al., 2017; Vernillo et al., 2020b). The longer aerial phase reflected a reduced need for active propulsion and a greater reliance on gravity to assist forward motion. Adjusting cadence was explored as a strategy to alter loading and energy expenditure (Vincent et al., 2019). Studies showed that running at a cadence within ±5% of the preferred value when going downhill minimized unit caloric expenditure and impulse loading, while a significantly lower cadence (−10%) increased heart rate and vertical impulse (Vincent et al., 2019).

The alterations in cadence, stride length, contact time and duty factor reflected the fundamental biomechanical strategies employed by runners to accommodate the varying demands of uphill and downhill terrains. These adaptations served specific functional purposes: the increased cadence and decreased aerial time during UR might be favourable for maintaining speed and reducing the energetic cost against the inclined surface, and reduced cadence and increased stride length during DR might allow runners to capitalize on gravitational assistance. Comprehending these baseline spatiotemporal adjustments provides the foundation for the interpretation of more complex kinetic and energetic adaptations. However, spatial-temporal parameters were most commonly analysed in the velocity range of 2.8–3.35 m/s and the gradient range of −11%–11%. The most challenging issue when selecting specific studies lies in the lack of consistency regarding the exact numerical values of gradient in the chosen protocols (Marszałek et al., 2024). Future studies require consideration of steeper gradients.

### 2.2 Ground reaction force and foot strike pattern

During UR, the normal GRF peaks, particularly the impact peak, tended to decrease compared with those during LR (Gottschall and Kram, 2005). This outcome was likely due to the more compliant landing strategy often adopted on inclines and the reduced vertical velocity at touchdown. Conversely, the parallel propulsive force peaks were considerably larger during UR, reflecting the increased effort required to push off and propel the body upward against gravity (Gottschall and Kram, 2005). At steeper uphill grades (+9°), normal impact force peaks were occasionally absent, and parallel propulsive peaks increased substantially (e.g., by 75% at +9°) (Gottschall and Kram, 2005) (Figure 1).

DR, which was characterised by increased normal impact force peaks and larger parallel braking force peaks, presented a different challenge compared with LR (Gottschall and Kram, 2005; Khassetarash et al., 2020; Vernillo et al., 2020b). The increased normal impact forces resulted directly from landing on a declining surface, which increased the vertical velocity component at touchdown. At a −9° slope, normal impact force peaks increased by 54%, and parallel braking force peaks by 73% compared with LR (Gottschall and Kram, 2005) (Figure 1). These elevated impact forces during DR have been a major concern in overuse injuries, particularly those related to impact loading (Giandolini et al., 2016a; Giandolini et al., 2015; Gottschall and Kram, 2005; Chan et al., 2021; Selvakumar et al., 2023), and increased vertical GRF loading rate has been identified as a biomechanical risk factor for overuse injuries (e.g., patellofemoral pain syndrome and plantar fasciitis) in distance runners (Willwacher et al., 2022). Vertical maximum accelerometer impacts exceeding 6G increased considerably with the negative slope during DR (Bascuas et al., 2021). Parallel braking forces were larger because the runner had to actively decelerate the body’s forward motion, which was accelerated by gravity.

We emphasize how slope altered foot strike pattern selection and, in turn, GRF characteristics. Empirically, increasing uphill slope shifted runners toward mid/forefoot striking, whereas a decreased downhill slope increased rearfoot striking (Giandolini et al., 2017; Horvais and Giandolini, 2013; Selvakumar et al., 2023; Vernillo et al., 2017). This slope-driven shift helped to explain the GRF patterns reported above: more anterior contacts on uphill reduced or even eliminated the normal impact peak, while the greater prevalence of rearfoot contacts on downhill, combined with higher vertical touchdown velocity contributed to larger normal impact and parallel braking peaks (Gottschall and Kram, 2005; Kowalski and Li, 2016).

### 2.3 Joint kinematics and kinetics

Running on various grades considerably altered the movement patterns and forces/moments experienced at the lower limb joints (hip, knee, and ankle) (Hirschman et al., 2024; Khassetarash et al., 2020; Lu et al., 2024; Padulo et al., 2012; Sundström et al., 2021; Vernillo et al., 2017; Vernillo et al., 2020b). These kinematic and kinetic adaptations reflected the changing demands for energy generation and absorption. Energy absorption and energy generation were calculated respectively across the entire stance phase. The normalized energy generation/absorption value for each joint was derived by calculating the total energy generation/absorption required per meter of running distance (normalized to step length). However, the trends in energy absorption and generation aligned with those of normalized energy absorption and generation (Khassetarash et al., 2020).

During UR, lower limb muscles performed a higher net mechanical work compared with LR and DR to increase the body’s potential energy (Vernillo et al., 2017). This increased demand for work was met by an increase in power output at all joints, particularly the hip (Vernillo et al., 2017). Kinematically, UR was often characterized by increased hip flexion and extension range of motion during stance, increased knee flexion, and increased ankle plantarflexion at touchdown and throughout stance to facilitate propulsion against the incline (Khassetarash et al., 2020; Padulo et al., 2012; Sundström et al., 2021; Vernillo et al., 2020b). The ankle joint’s contribution to theoretical leg stiffness increased with steeper uphill slopes, whereas the knee’s contribution decreased, particularly during the first half of stance (Hirschman et al., 2024). Also, the ankle contributed 55% at level to 46% at +5.71° uphill of total positive power, while the hip contribution increased from 28% at level to 36% at +5.71° uphill at the speed of 2.25 m/s (Nuckols et al., 2020). As hip contribution raised, the ankle remained the dominant source of positive mechanical power during UR as the slope increased. These findings suggested that UR involved enhanced hip activity with the ankle continuing to serve as the principal joint for propulsion while the hip supplemented the required positive work. Fatigue during prolonged graded running led to altered joint kinematics, such as less extended knee and hip joints in the swing phase and less extended knee in stance during uphill sections, indicating changes in energy generation and absorption strategies (Genitrini et al., 2024).

By contrast, DR was primarily characterized by energy dissipation rather than generation, as gravity provided assistance to forward motion (Vernillo et al., 2017). Controlling the descent and absorbing impact forces were hallmark features of DR biomechanics. Kinematically, DR typically involved greater knee flexion at touchdown and maximal knee flexion during the stance phase, especially on steeper downhill slopes (Khassetarash et al., 2020; Sundström et al., 2021; Vernillo et al., 2020b). The ankle joint tended to be more dorsiflexed at initial contact, particularly with a rearfoot strike pattern, which was more common downhill (Giandolini et al., 2017; Horvais and Giandolini, 2013; Vernillo et al., 2017). Kinetically, studies reported increased energy absorption and peak negative power at the ankle and knee during DR compared with LR (Khassetarash et al., 2020; Vernillo et al., 2020b). Specifically, the knee joint showed significant increases in peak joint moments, peak powers and energy absorption on steeper downhill grades, and the ankle exhibited reductions in peak moments and powers but increased energy absorption (Khassetarash et al., 2020; Vernillo et al., 2020b). The contribution of the knee joint to theoretical leg stiffness increased with steeper downhill slopes, particularly during the first half of stance, while the ankle’s contribution decreased, which reflecting the increased role of knee extensors in impact absorption and descent control (Hirschman et al., 2024).

## 3 Physiological and neuromuscular responses to graded running

**FIGURE 2:**
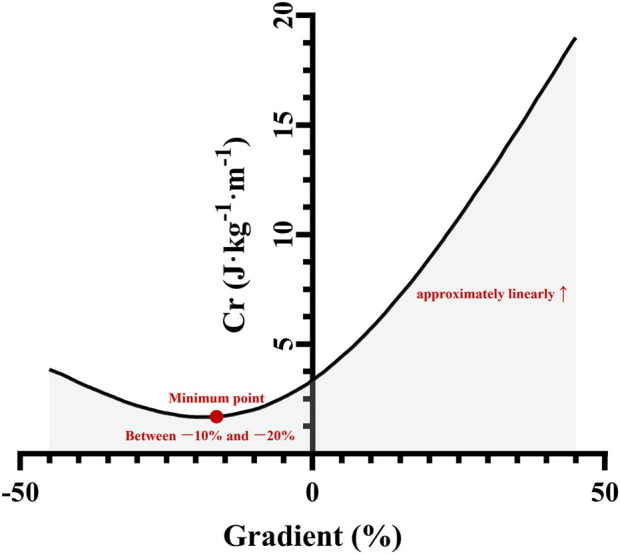
The metabolic energy cost of running (Cr) as a function of the gradient. Adapted and based on data from Minetti et al. (2002).

**FIGURE 3:**
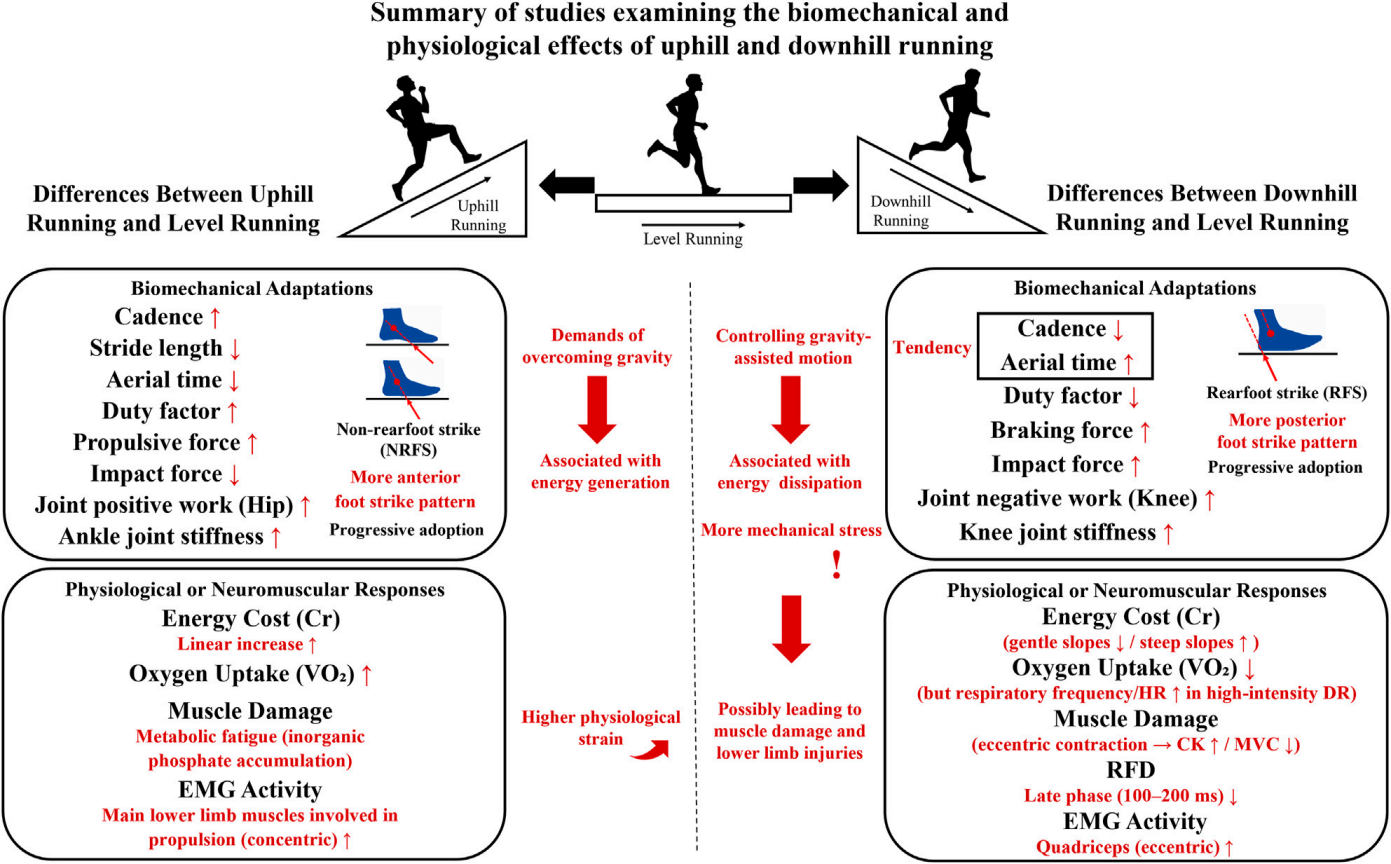
Summary of studies examining the biomechanical and physiological effects of uphill and downhill running. Note: All variable comparisons were made at the same running speed, excluding extreme steep slopes to ensure data comparability. ↑,↓ indicate increase and decrease in biomechanical and physiological variables, based on the review by Giandolini et al. (2016b), Vernillo et al. (2017) and Bontemps et al. (2020), and other previous research findings. HR: Heart Rate, CK: Creatine Kinase, MVC: Maximal Voluntary Contraction, RFD: Rate of Force Development, EMG: Electromyography, DR: Downhill Running.

In addition to mechanical changes, running on inclines and declines elicited distinct physiological and neuromuscular responses, particularly regarding energy expenditure (Figure 2), cardiorespiratory demands and muscle activity, fatigue and damage (Figure 3) (Bascuas et al., 2021; Bontemps et al., 2020; Garnier et al., 2020; Giandolini et al., 2017; Giandolini et al., 2016b; Lemire et al., 2023; Lemire et al., 2018; Lemire et al., 2021; Vernillo et al., 2017).

### 3.1 Energy cost and cardiorespiratory responses

The energy cost of running (Cr), often expressed as oxygen uptake (VO_2_) per unit mass and distance (e.g., mL/kg/km or J/kg/m), is a key determinant of endurance performance and profoundly affected by surface gradient (Vernillo et al., 2017). During UR, Cr increased approximately linearly with positive slope, reflecting the increased mechanical work required to lift the body’s center of mass against gravity (Figure 2) (Bascuas et al., 2021; Garnier et al., 2020; Lussiana et al., 2013; Vernillo et al., 2017). This increased metabolic demand was associated with higher VO_2_, carbon dioxide production (VCO_2_), pulmonary ventilation (VE), and heart rate (HR) (Bascuas et al., 2021; Garnier et al., 2020; Lemire et al., 2018; Lemire et al., 2021; Minetti et al., 2002).

DR presented a distinct metabolic profile. Studies revealed that Cr decreased with negative slope until a minimum was reached, typically between −10% and −20%, after which it increased again on steeper declines (Figure 2) (Lussiana et al., 2013; Vernillo et al., 2017; Minetti et al., 2002). This initial decrease occurred due to gravity assisting forward motion, which reduced the need for active propulsion. However, on steeper downhill grades, the metabolic cost increased again, possibly due to the greater muscular effort required for braking, impact absorption and stabilisation (Bascuas et al., 2021; Gottschall and Kram, 2005; Vernillo et al., 2017). During DR, peak VO_2_ (the highest VO_2_ obtained on the test), VCO_2_, VE, and maximum HR were significantly decreased compared with those during UR, even at similar speeds or metabolic intensities (Bascuas et al., 2021; Garnier et al., 2020; Lemire et al., 2018; Lemire et al., 2021). DR was associated with lower VO_2_ and Cr compared with LR and UR (Garnier et al., 2020). At the same running speed (6 km/h), heart rate and respiratory frequency were lower in DR compared with UR. However, high-intensity DR, even at submaximal metabolic intensity (e.g., 70% VO_2max_), still led to exacerbated HR and respiratory frequency, potentially reaching maximal values, highlighting the unique cardiorespiratory stress despite lower oxygen consumption (Lemire et al., 2021). These findings highlighted the need for cautious interpretation of cardiorespiratory data as markers of exercise intensity during DR, particularly for training load prescription (Garnier et al., 2020; Lemire et al., 2018).

However, the relationship between biomechanics and energy cost differs between grades (Lemire et al., 2023). During UR, contact time (*r* = −0.54, *p* = 0.017) and duty factor (*r* = −0.57, *p* = 0.010) were negatively correlated with Cr, and aerial time (*r* = 0.57, *p* = 0.011), mass-specific peak vertical GRF (*r* = 0.55, *p* = 0.012) and positive external work showed a positive correlation (*r* = 0.49, *p* = 0.035). Meanwhile, DR showed no significant associations with these variables (Lemire et al., 2023). Consistent with the mechanistic interpretation, positive external work accounted for ∼69% of total external work in UR (vs. ∼31% negative work), and economical runners in UR exhibited step frequency of 2.75 ± 0.10 Hz versus 2.86 ± 0.10 Hz (≈4% lower; *p* = 0.026) in addition to a ∼7% longer contact time (0.30 ± 0.02 s vs. 0.28 ± 0.02 s; *p* = 0.013) and ∼4% longer step length (1.01 ± 0.04 m vs. 0.97 ± 0.03 m; *p* = 0.038). Altogether, these data support that optimization of stance duration and work distribution during UR was associated with lower Cr, whereas DR economy at the tested speed/slope was less directly explained by single spatiotemporal/GRF variables (Lemire et al., 2023).

### 3.2 Muscle fatigue and damage during downhill running

DR was particularly well-known for inducing considerable muscle fatigue and exercise-induced muscle damage (EIMD) due to the high volume of eccentric muscle contractions required for braking and shock absorption (Bontemps et al., 2025; Bontemps et al., 2020; Coratella et al., 2024; Giandolini et al., 2017; Giandolini et al., 2016b; Khassetarash et al., 2023; Khassetarash et al., 2022; Lima et al., 2021; Varesco et al., 2022). EIMD was often assessed through measures such as delayed onset muscle soreness (DOMS), increases in serum creatine kinase (CK) concentration, reductions in maximal voluntary contraction (MVC) force, and alterations in muscle function like the rate of force development (RFD) (Bontemps et al., 2025; Bontemps et al., 2020; Coratella et al., 2024; Khassetarash et al., 2023; Khassetarash et al., 2022; Lemire et al., 2021; Varesco et al., 2022).

The magnitude of EIMD and fatigue in response to DR was influenced by main factors such as slope, exercise duration and running speed. Following DR exercise induced muscle damage, running biomechanics impairment might persist for up to 48 h (Markus et al., 2025). In general, steeper slopes, longer durations, and higher speeds resulted in more pronounced effects (Bontemps et al., 2020). Individuals who were less accustomed to DR typically experienced a greater muscle damage (Bontemps et al., 2020). Whether manipulated independently or not, altering the DR characteristics might result in varying extents of EIMD. However, significant variations existed in research protocols employing the DR model, making it challenging to definitively attribute the primary factors responsible for muscle damage and fatigue in DR. Additional high-quality studies are required, employing a consistent methodological approach when evaluating muscle damage (Bontemps et al., 2020).

In addition, researchers observed repeated bout effect (RBE), whereby prior exposure to eccentric exercises such as DR led to reduced muscle soreness, smaller increases in CK, less loss of MVC force and less decline in voluntary activation during subsequent sessions (Bontemps et al., 2025; Bontemps et al., 2020; Khassetarash et al., 2023; Khassetarash et al., 2022). This adaptation involved changes in neural drive and biomechanical adjustments, such as attenuated increases in duty factor and less reduction in leg and joint quasi-stiffness during the second DR bout compared with the first DR bout (Khassetarash et al., 2023; Khassetarash et al., 2022). However, although repeated bouts reduced perceived muscle soreness, they did not necessarily mitigate neuromuscular fatigue responses, such as MVC loss or peripheral/central fatigue indicators, suggesting distinct physiological adaptations (Bontemps et al., 2025; Khassetarash et al., 2022). Additionally, a previous study indicated that the RBE after DR was characterized by changes to central and global fatigue parameters and running biomechanics without substantially changing the Cr (Khassetarash et al., 2022). Despite the lower cardiorespiratory demands, high-intensity DR could still exacerbate muscular fatigue compared with UR at a similar metabolic intensity, indicating that muscular stress, particularly eccentric loading, might be the primary limiting factor during intense descents, rather than cardiorespiratory capacity (Lemire et al., 2021). The mechanisms of the observed effects of repeated bout on running biomechanics remain incompletely clarified (Khassetarash et al., 2023).

Compared with UR, high-intensity DR exacerbated muscular fatigue, leading to greater reductions in the torque of the knee and hip extensors (Lemire et al., 2021). After DR, the maximum voluntary contraction (MVC) force/torque of knee extensors and plantar flexors typically decreased significantly, with specific reductions ranging from −14% to −55% for knee extensors and −15% to −25% for plantar flexors (Bontemps et al., 2020). Although prolonged hilly running (combined uphill and downhill) displayed similar patterns of neuromuscular fatigue and etiology to LR, “pure” DR exhibited specific fatigue-related features, including severe tissue damage and low-frequency fatigue (excitation–contraction coupling failure) attributed to mechanical stress (Giandolini et al., 2017; Giandolini et al., 2016b). By contrast, low-frequency fatigue in high-intensity UR was more likely related to inorganic phosphate accumulation (Giandolini et al., 2017; Giandolini et al., 2016b).

DR could impair the muscle’s capacity to produce maximum force (MVC) and a runner’s overall ability to rapidly develop force (RFD) (Coratella et al., 2024; Varesco et al., 2022). Specifically, DR affected the late phase (e.g., 100–200 ms) of RFD but not the early phase (e.g., 0–50 ms) (Coratella et al., 2024; Varesco et al., 2022). This outcome suggested that impairments in rapid force-generating capacity following DR were likely located within the skeletal muscle itself, potentially due to reduced muscle-tendon stiffness or issues with the muscle contractile apparatus, rather than alterations in the neural mechanisms underlying early RFD (Varesco et al., 2022). The late phase of RFD might serve as an additional marker of muscle damage in trail running (Coratella et al., 2024). These neuromuscular alterations, including reductions in MVC and RFD, persisted for several days following a bout of DR (Coratella et al., 2024; Lima et al., 2021; Varesco et al., 2022).

Although DR could cause considerable muscle damage and fatigue, the decrease in running economy following DR was not strongly linked to reductions in force production capacity (MVC) (Lima et al., 2021). This finding indicated that strength loss alone might not have been the main factor influencing running economy after DR. which implied that other mechanisms probably contribute to the impaired economy. Strategies for mitigating the effects of DR-induced fatigue and damage remained an active area of research. Prior exposure to DR was considered one of the most effective preventive measures (Bontemps et al., 2020). *In-situ* methods, such as the use of lower-limb compression garments, which might reduce soft tissue vibrations, showed potential benefits for acute neuromuscular responses; however, their long-term effectiveness requires further confirmation (Bontemps et al., 2020; Gassier et al., 2025). Adjustment of running kinematics, including changes to foot strike pattern or cadence, was also explored (Giandolini et al., 2016b; Vernillo et al., 2020a; Vincent et al., 2019). Although anterior foot strike patterns might redistribute eccentric work between the knee extensors and plantar flexors, which potentially affected damage severity in these muscle groups; in addition, studies examining the effect of foot strike pattern or deliberate switching during prolonged graded running did not demonstrate remarkable benefits for overall neuromuscular fatigue (Giandolini et al., 2017; Vernillo et al., 2020a, 2024). These findings suggested that the ability to adapt foot strike to changing terrain, rather than adhering to any specific pattern, might be more important to reduce fatigue (Vernillo et al., 2020a).

### 3.3 Muscle activity

The pattern and intensity of muscle activation, as measured by electromyography (EMG), also adapted to the demands of graded running. These adaptations reflected changes in how different muscle groups generated power, absorbed energy, and stabilized the joints.

During UR, a general increase was observed in the activity of the main lower-limb muscles involved in propulsion, such as the gastrocnemius, soleus, vastus lateralis, rectus femoris, gluteus maximus and hamstrings, compared with LR (Swanson and Caldwell, 2000; Vernillo et al., 2020b). Higher-average EMG amplitudes in the gastrocnemius, soleus, rectus femoris, vastus lateralis and gluteus maximus during the stance phase of high-speed incline running was reported (Swanson and Caldwell, 2000). Compared with LR, the EMG activity of the lower limb muscle was 6% greater during UR at 10% grade (Vernillo et al., 2017). UR required a greater activation of the vastus group (+23%) and soleus (+14%) accompanied by reduced activation of the semitendinosus (−17%), gracilis (−18%) and rectus femoris (−29%) compared with LR (Vernillo et al., 2017). The increased activity in these muscles partially supported the greater positive work and power generation required for ascent as the findings mentioned earlier (Section 2.3). However, a previous study reported that types of synergy were consistent between treadmill LR and UR, suggesting that basic patterns of locomotor muscle activity were consistent across running speeds (2.5, 3.3, and 4.1 m/s) for each condition (level and 10% grade) (Saito et al., 2018).

DR involved a shift in muscle function towards eccentric contractions, particularly in the knee extensors (quadriceps), to control the rate of descent and absorb impact energy (Giandolini et al., 2016b; Vernillo et al., 2020b). Although some studies reported no significant changes in vastus lateralis activity compared with LR at moderate downhill grades (Vernillo et al., 2020b), others suggested variations in the patterns depending on the phase of the gait cycle and specific muscle examined. The effects of foot strike pattern on muscle activity during DR was investigated, and the results revealed the association of anterior (forefoot) strike patterns with higher gastrocnemius lateralis activity and lower tibialis anterior and vastus lateralis activity (Giandolini et al., 2017). This finding indicated that the manner by which runners managed impact and energy dissipation during DR influenced the patterns of muscle recruitment.

The distinct muscle activation patterns observed in UR and DR underscored their various physiological demands. UR emphasised concentric work and power generation, resulting in an increased activity of the propulsive muscles. By contrast, DR emphasised eccentric work and energy absorption, particularly in the quadriceps, which contributed to the muscle damage and soreness commonly associated with DR (Bontemps et al., 2020; Giandolini et al., 2016b). More systematic studies are needed in the future, especially those using consistent protocols for slope, speed, and muscle group selection, to comprehensively cover the changes in each muscle at different slopes. Furthermore, future research should focus on the synergistic actions of muscles during LR, UR and DR, to better understand how muscle interactions influence performance and biomechanics.

The summary of studies examining the biomechanical and physiological effects of UR and DR is presented in Figure 3.

## 4 Factors influencing graded running biomechanics and physiology

To avoid conflating slope-driven adaptations (treated in Sections 2 and 3) with modulating variables, this section focuses on how each factor alters responses given a certain slope (i.e., interaction with grade). For each factor, we initially state a main (grade-independent) effect and then detail the interaction with grade (UR and DR). Factors, including footwear, foot strike pattern, individual runner characteristics, pacing strategy, cadence and running speed, modulate the biomechanical and physiological responses to UR and DR. Understanding the influence of these factors is crucial for the performance optimisation and minimisation of injury risk (Table 1).

**TABLE 1:** Summary of factors influencing graded running biomechanics and physiology.

Factor	Grade-independent effect	Interaction with uphill running (UR)	Interaction with downhill running (DR)	References
Footwear	• Carbon-plated shoes ↓ Cr vs. traditional shoes (all conditions) • Minimal shoes ↓ metabolic cost vs. traditional shoes, independent of slope	• Metabolic savings with carbon-plated shoes may be slightly less on grades vs. LR • High LBS shoes ↑ efficiency and knee load	• MAX footwear ↑ biomechanical loading	Hunter et al. (2022), Whiting et al. (2022), Lussiana et al. (2013), Lu et al. (2024), Chan et al. (2018)
Foot Strike Pattern	• FFS ↓ peak normal impact force vs. RFS; may eliminate impact peak	• ↑ Prevalence of FFS. • ↓ Tibial loading	• ↑ RFS prevalence • FFS ↓ vertical impact • Gait retraining effectiveness inconsistent (outdoor vs. treadmill) • Deliberate switching → no improvement in neuromuscular fatigue	Lieberman et al. (2010), Kowalski and Li (2016), Giandolini et al. (2017), Horvais and Giandolini (2013), Zhang et al. (2019), Vernillo et al. (2020a), 2024, Chan et al. (2021)
Runner Characteristics	• Elite trail runners ↓ Cr vs. experienced trail runners • Elite trail runners ↑ max torque/power vs. road runners • Cr strongly inter-correlated across gradients	• Elites maintain ↓ contact time vs. amateurs • ↑ Hip power → ↓ Cr	• RBE ↓ muscle damage/soreness/fatigue & alters biomechanics • High-level runners → midfoot strike; Medium-level → RFS • Trail runners ↑ vertical loading rate & foot strike angle	Besson et al. (2023), Sabater Pastor et al. (2023), Breiner et al. (2019), Padulo et al. (2012), Bontemps et al. (2025), Khassetarash et al. (2022), 2023, Lussiana et al. (2013), Selvakumar et al. (2023)
Cadence	• ↑ Cadence ↓ per-step peak load, impulse, and cumulative tissue damage (patellofemoral joint, tibia, Achilles tendon) across speeds/gradients	• Primary speed modulator (↑ cadence > ↑ stride length)	• Cadence ±5% preferred ↓ caloric cost & impulse loading • Cadence ↓ −10% → ↑ HR & vertical impulse	Vincent et al. (2019), Van Hooren et al. (2024)
Speed	• ↑ Speed consistently → ↓ contact time and ↑ joint energy generation/absorption, cadence, and Cr across gradients	• Disproportionate ↑ parallel GRF & positive joint work (hip) • Priority on stride frequency → ↓ knee power gen & ↓ ankle energy gen • Linear ↑ tibial/Achilles loading • Linear ↑ Cr	• Quadratic ↑ vertical impact peaks & braking forces • ↑ Patellofemoral damage (↑ eccentric loading) • ↑ Braking impulse & normal loading rate • Steep grades blunt ↑ knee energy abs → load shift ankle/hip • Attenuated ↑ Cr until gradients > −15%	Khassetarash et al. (2020), Vernillo et al. (2020b), Padulo et al. (2012), Gottschall and Kram (2005), Van Hooren et al. (2024), Sundström et al. (2021), Lemire et al. (2023), Rice et al. (2024)

↑, Increase; ↓, Decrease; →, Leads to; Cr, Cost of Running; LBS, Longitudinal Bending Stiffness; RBE, Repeated Bout Effect; GRF, Ground Reaction Force; FFS, Forefoot Strike; RFS, Rearfoot Strike; MAX, Maximalist footwear; HR, Heart Rate; LR, Level Running.

### 4.1 Footwear

For the grade-independent effect, the design of running footwear, particularly features such as cushioning, midsole compliance and the presence of carbon-fiber plates (e.g., Nike Vaporfly 4%) showed significant metabolic savings or benefit compared with traditional racing shoes across all conditions (LR, UR and DR) (Whiting et al., 2022; Hunter et al., 2022). Minimal shoes also offered a slight metabolic advantage over traditional shoes during LR, UR and DR, potentially due to differences in shoe mass and kinematic alterations (Lussiana et al., 2013).

A biomechanical study investigated the effects of maximalist footwear (MAX) versus traditional running shoes on impact loading during treadmill running; it suggested that MAX failed to attenuate impact forces during DR and might paradoxically exacerbate biomechanical loading (Chan et al., 2018). The longitudinal bending stiffness (LBS) of running shoes also impacted biomechanics, particularly during transitions between grades. High LBS shoes might improve efficiency during uphill transitions by altering joint work distribution but could potentially increase knee joint loading (Lu et al., 2024). This phenomenon highlighted the complex interplay among shoe design, terrain and biomechanical outcomes, suggesting that optimal footwear characteristics varied depending on specific running conditions.

Modular footwear designed to partially offset treadmill grade had been explored in walking conditions. A previous study revealed that shoes with a slight inclination minimised the metabolic cost of walking on downhill and uphill grades, suggesting that footwear could potentially assist locomotion on rolling terrain (Antonellis et al., 2020). Although this study focused on walking, it raised the possibility of developing footwear specifically tailored to graded running.

More recent research focused on the effect of highly cushioned shoes with embedded carbon-fiber plates (Hunter et al., 2022; Whiting et al., 2022). Although one study suggested that the metabolic savings might have been slightly less on grades compared with LR, another discovered the metabolic benefits to be similar across uphill, level and downhill conditions (Hunter et al., 2022; Whiting et al., 2022). However, given inconsistencies in shoe types, slopes and speed selections, the interactions of such footwear metabolic savings under sloped conditions still remain unclear. Additionally, a study found that increasing LBS in trail running footwear, particularly through the use of carbon plates, did not improve running economy during LR but led to a slight performance degradation during UR, with a 2% increase in metabolic energy expenditure. The benefits of increased LBS may not apply to technical trail running, especially when running at slower speeds or on unstable terrains, highlighting the importance of considering the specific context when designing footwear for different running environments (Jaboulay and Giandolini, 2025). Another research also showed that specific trail running shoes might fail to mitigate trail running injuries (Hamill et al., 2022). Thus, based on the findings of existing studies, more research is needed to provide theoretical support for the supportive technologies of graded running or trail running shoes.

### 4.2 Foot strike pattern

Foot strike pattern (rearfoot, midfoot or forefoot strike) is a modifiable aspect of running gait that influences biomechanics and loading (Lieberman et al., 2010). For grade-independent effect, the forefoot strike pattern tended to reduce the peak normal impact force (impact peak might not also be visible) compared with the rearfoot strike pattern (Kowalski and Li, 2016; Lieberman et al., 2010). However, deliberately switching foot strike pattern during prolonged graded running caused no improvement on neuromuscular fatigue outcomes, and perhaps no universal “best” foot strike pattern had emerged (Vernillo et al., 2024).

A previous study suggested the possibility of using forefoot striking might be a good strategy to reduce impacts, especially during DR (Kowalski and Li, 2016). However, the effectiveness of gait retraining to modify foot strike pattern and reduce impact loading during sloped running conditions showed inconsistency (Zhang et al., 2019). Following completion of an indoor treadmill-based gait retraining program, 80% of participants achieved substantial reductions in peak tibial shock during level and sloped running conditions on a treadmill. However, among these training-responsive runners, reductions in peak tibial shock were maintained during outdoor LR but not during outdoor slope running (Zhang et al., 2019). Therefore, the difference in the regulatory effects of foot strike patterns between outdoor environments and treadmills still requires further evaluation.

Despite the potential for foot-strike manipulation to alter biomechanics, no considerable differences were observed in studies investigating the effect of deliberately switching foot strike patterns or maintaining a specific pattern on neuromuscular fatigue during prolonged graded running (Vernillo et al., 2020a, 2024). This finding suggested that the ability to adapt one’s technique to specific terrain and conditions, rather than adhering to a rigid pattern or switching strategy, was more important for minimising fatigue and optimising performance (Vernillo et al., 2020a; Giandolini et al., 2015).

### 4.3 Runner characteristics and adaptation

Individual runner characteristics, such as fitness level, training history, experience with graded running, and competitive status (elite or recreational), influenced both biomechanical and physiological responses to LR, UR and DR (Besson et al., 2023; Bontemps et al., 2025; Bontemps et al., 2020; Breiner et al., 2019; Khassetarash et al., 2023; Khassetarash et al., 2022; Padulo et al., 2012; Sabater Pastor et al., 2023). For grade-independent effect, elite trail runners, compared with their experienced counterparts, showed a lower cost of running despite similar biomechanics; this finding was potentially linked to differences in neuromuscular characteristics, such as the power–torque–velocity profile (Besson et al., 2023). They also exhibited higher theoretical maximal torque and maximal power in the force–velocity profile compared with elite road runners, which might have been related to the specific strength requirements of graded running or training adaptations (Sabater Pastor et al., 2023). Running economy values across level, uphill and downhill grades were strongly intercorrelated (Breiner et al., 2019). This condition suggested that runners who were economical on level surfaces also tended to be economical on uphill and downhill grades, which implied that interindividual differences in running economy reflected intrinsic physiology and skill and that experienced runners were equally skilled across different grades (Breiner et al., 2019).

Elite runners exhibited more efficient biomechanical adjustments, such as various contact time responses to slope, compared with amateurs (Padulo et al., 2012). Experience with graded running, particularly prior exposure to DR, led to adaptation through RBE, which resulted in attenuated muscle damage, soreness and fatigue responses, and altered biomechanical strategies during subsequent bouts (Bontemps et al., 2025; Bontemps et al., 2020; Khassetarash et al., 2023; Khassetarash et al., 2022). This adaptation involved changes in neural drive and muscle function, which allowed runners to better tolerate the eccentric loading of DR (Bontemps et al., 2025; Khassetarash et al., 2022). During trail DR conditions, high-level trail runners (classified by skill in DR) adopted a midfoot strike pattern, but medium-level trail runners adopted a rearfoot strike pattern (Lussiana et al., 2013). However, trail runners, who frequently encountered downhill terrains, also experienced a greater vertical instantaneous loading rate and foot strike angle during DR compared with road runners, which might potentially contribute to their higher injury incidence (Selvakumar et al., 2023). Because prospective cohort studies found that trail runners experienced 10.7 to 19.6 running-related injuries per 1,000 h of training (mainly affecting the knee, shin/lower leg, and foot/toes), whereas road runners sustained only 2.5 to 5.8 injuries per 1,000 h of running (Selvakumar et al., 2023).

Specific training programs targeting graded running were likely necessary to optimise performance and adaptation to the unique demands of uphill and downhill terrains (Gentilin et al., 2023; Lemire et al., 2023; Lemire et al., 2021). Strength training was suggested for lower-level trail runners to improve Cr, potentially by enhancing their ability to manage forces on varied terrain (Besson et al., 2023). High-intensity training, including uphill and downhill variations, was effectively maintained an exercise performance despite a reduction in overall training volume (Gentilin et al., 2023).

### 4.4 Cadence

Manipulating cadence has been explored as a strategy to modify loading and energy cost (Vincent et al., 2019; Van Hooren et al., 2024; Vernillo et al., 2020b). Increasing cadence generally reduced per-step peak load and impulse and cumulative tissue damage at common injury-prone sites (patellofemoral joint, tibia and Achilles tendon) across various speeds and gradients, suggesting that it could be an effective strategy for mitigating tissue loading (Van Hooren et al., 2024). Running at cadences slightly different from one’s preferred rhythm (e.g., ±5%) minimised caloric unit cost and impulse loading, whereas larger deviations (e.g., −10%) tended to increase physiological and mechanical stress, particularly during DR (Vincent et al., 2019). During LR, a slight increase in cadence enables faster running speeds, whereas during UR and DR, faster running speeds are associated with a considerable increase in cadence (Vernillo et al., 2020b). Therefore, in graded running, the strategy of adjusting and maintaining the cadence is crucial.

### 4.5 Running speed

Running speed, which exhibited complex interactions with surface gradient, constituted a critical modulator of biomechanical and physiological responses during graded running (Khassetarash et al., 2020; Vernillo et al., 2020b). However, as velocity increased, biomechanical and physiological parameters demonstrated consistent directional changes across gradients: contact time decreased, and joint energy generation/absorption, cadence and Cr increased (Padulo et al., 2012; Sundström et al., 2021; Vernillo et al., 2017; Khassetarash et al., 2020; Vernillo et al., 2020b). Yet, the magnitude and mechanistic drivers of these adaptations showed substantial divergence among UR, DR and LR.

During UR, elevated speeds amplified propulsive demands, resulting in disproportionate increases in parallel GRF and positive joint work—particularly at the hip (Vernillo et al., 2017). A high speed (4.17 m/s) was achieved by prioritizing stride frequency over stride length, and it reduced vertical displacement per step. This finding suppressed knee peak power generation despite higher speeds and decreased ankle energy generation and positive parallel impulse (Khassetarash et al., 2020; Vernillo et al., 2020b). In addition, cumulative loading at the tibia and Achilles tendon escalated linearly with speed, which heightened the risk of bone stress injuries and tendinopathy (Rice et al., 2024; Van Hooren et al., 2024).

By contrast, DR exhibited quadratic increases in vertical impact peaks (e.g., +54% at −9° slope compared with LR) and braking forces under faster conditions (Gottschall and Kram, 2005). This condition accelerated patellofemoral joint damage due to exacerbated eccentric loading of the knee extensors, with tissue stress models predicting over 40% higher cumulative damage at 5 m/s versus 3 m/s on −10% slopes (Van Hooren et al., 2024). Moreover, high speed escalated braking impulse and normal loading rate, but steep grades (−10°) blunt speed-induced increased in knee energy absorption, which shifted the load to ankle and hip joints (Khassetarash et al., 2020; Vernillo et al., 2020b). In addition, although EMG showed comparable u-shaped curves across the grades tested, an interaction between running speed and grade only occurred in tibialis anterior and vastus lateralis (Vernillo et al., 2020b). Therefore, similar muscle recruitment patterns might still result in different kinetic variables and external forces (Khassetarash et al., 2020; Vernillo et al., 2020b).

Metabolically, Cr showed a linear increase with speed across all gradients; however, the slope-dependent efficiency profile persisted: UR maintained high Cr sensitivity to velocity, whereas DR displayed attenuated Cr increases until steep gradients (greater than −15%), where braking demands dominated (Lemire et al., 2023; Vernillo et al., 2017).

Overall, ankle, knee and hip joint kinetics, spatiotemporal parameters, ground reaction forces and muscle activations were significantly influenced by running speed and grade. Speed and grade interact for various joint kinetic variables, but the effect of grade on joint kinematics was not modulated by speed (Khassetarash et al., 2020; Vernillo et al., 2020b). Thus, while increasing speed uniformly intensified biomechanical stressors, its interaction with gradient dictated distinct risk profiles: UR primarily amplified propulsion-phase risks through elevated tibial and Achilles tendon loading, whereas DR exacerbated impact-phase hazards via heightened patellofemoral joint stress and knee microtrauma. Importantly, cadence manipulation served as a key strategy for speed modulation. Consequently, training prescriptions required gradient-specific velocity thresholds combined with cadence optimisation, which specifically maintained cadence within ±5% of preferred during DR, to mitigate tissue overload during high-intensity graded running (Rice et al., 2024; Van Hooren et al., 2024; Vincent et al., 2019).

In summary, the biomechanical and physiological responses to UR and DR are influenced by various modulating factors and exhibit significant differences. However, these factors also exert a number of grade-independent effects. Additional research is needed to systematically explore the interactive effects of these modulating factors and grade on biomechanical and physiological responses.

## 5 Methodological considerations and future directions

Investigation of the biomechanics of UR and DR presents unique methodological challenges compared with LR. To date, most research had been conducted on treadmills or ramps, which offered controlled environments for the manipulation of speed and slope and collection of precise kinematic and kinetic data (Chan et al., 2021; Gottschall and Kram, 2005; Hirschman et al., 2024; Honert et al., 2022; Hunter et al., 2022; Khassetarash et al., 2020; Lu et al., 2024; Padulo et al., 2012; Sundström et al., 2021; Vernillo et al., 2020b; Whiting et al., 2022). Previous studies showed that graded running biomechanics were highly comparable between treadmill and overground running (Firminger et al., 2018; Van Hooren et al., 2020), but it was also reported that gait retraining effects observed on a treadmill might not fully translate to graded running on the ground or in the real world (Zhang et al., 2019). Treadmill or ramp running might not have fully replicated the complex and variable terrain encountered in real-world trail running, which involved uneven surfaces, obstacles and unpredictable changes in gradient and direction (Genitrini et al., 2024; Hamill et al., 2022; Vincent et al., 2022). Wearable sensors and advanced modelling techniques have increasingly been used to estimate GRF from plantar pressure data during graded running; their application provided promising opportunities for real-world biomechanical assessment outside the laboratory (Alcantara et al., 2022; Honert et al., 2022). Field studies using wearable sensors were increasingly being employed to capture biomechanical data in ecological conditions and provided valuable insights into how runners adapted to real-world terrain despite the challenges remaining in data accuracy and analysis compared with laboratory settings (Alcantara et al., 2022; Chan et al., 2021; Genitrini et al., 2024; Giandolini et al., 2015; Honert et al., 2022; Zhang et al., 2019).

Despite the growing body of literature, several areas warrant further investigation. More studies on the interactive effects of modulating factors and grade on biomechanical and physiological responses are still needed, especially regarding footwear characteristics, habitual foot strike patterns and other neglected factors (Hamill et al., 2022; Kowalski and Li, 2016; Lu et al., 2024; Vernillo et al., 2020a; Whiting et al., 2022). The long-term effects of different training strategies, including gait retraining, strength training, high-intensity training and specific graded running protocols, on biomechanical adaptations, injury risk and performance in UR and DR require more research (Besson et al., 2023; Gentilin et al., 2023; Sabater Pastor et al., 2023; Vincent et al., 2022; Zhang et al., 2019; Zhang et al., 2021). The specific mechanisms underlying the cardiorespiratory responses to high-intensity DR and their implications for training prescription warrant further investigation (Garnier et al., 2020; Lemire et al., 2018; Lemire et al., 2021). Finally, prospective studies should be conducted to confirm the identified injury risk factors and evaluate the effectiveness of proposed prevention strategies in real-world trail running populations (Viljoen et al., 2022; Viljoen et al., 2021; Vincent et al., 2022). The use of advanced modelling techniques, such as estimating internal loading from external measures, holds promise for an improved comprehension of tissue-level stress during graded running in various conditions (Honert et al., 2022; Rice et al., 2024; Ye et al., 2024).

Compared with LR, UR exhibits increased cadence and duty factor, shortened stride length and aerial time and a shift toward mid/forefoot strike patterns. GRF shows considerably higher propulsive peaks with diminished vertical impact, and joint kinetics are dominated by hip power output and increased ankle quasi-stiffness contribution. Physiologically, UR causes the linear increase in energy cost linearly with slope, accompanied with increased VO_2_, HR and concentric muscle activation. Differently, DR demonstrates reduced duty factor and higher rearfoot strike prevalence. GRF feature amplifies vertical impact and braking force peaks, and the knee joint increases energy absorption and quasi-stiffness contribution. Physiologically, DR minimises Cr between −10% and −20% gradients but increases on steeper declines. Despite a lower oxygen consumption, high-intensity DR exacerbates HR responses and induces elevated CK, reduced MVC and impaired late-phase RFD (100–200 ms).

Equipment properties, technical strategies and individual adaptability modulate graded running responses through dual mechanisms: grade-independent effect (e.g., universal energy reduction by carbon-plated shoes; decreased tissue loading via cadence increase) and interactions with grade (e.g., heightened DR impact with MAX; vertical impact force attenuation by forefoot strike especially during DR; impaired DR performance when uphill energy expenditure exceeds 10.4% above average). More research in the future is needed to systematically explore the interactions between these modulating factors and slope.

In conclusion, graded running imposes distinct biomechanical and physiological adaptations. Footwear properties, foot strike patterns, individual adaptability, cadence, speed and pacing strategies collectively modulate these responses in some aspects. Future research should prioritise multifactorial–slope interactions in real-world settings, optimisation of slope-specific training, improvement of methods to prevent injuries and applications of wearable technology.
